# Orexin/hypocretin system dysfunction in patients with Takotsubo syndrome: A novel pathophysiological explanation

**DOI:** 10.3389/fcvm.2022.1016369

**Published:** 2022-11-03

**Authors:** Rajna Knez, Milan Niksic, Elmir Omerovic

**Affiliations:** ^1^Gillberg Neuropsychiatry Centre, Department of Psychiatry and Neurochemistry, Institute of Neuroscience and Physiology, Sahlgrenska Academy, University of Gothenburg, Gothenburg, Sweden; ^2^Research and Development, Department of Women's and Child Health, Skaraborg Hospital, Skövde, Sweden; ^3^Institution for Health, School of Health Sciences, University of Skövde, Skövde, Sweden; ^4^Department of Cardiology, Skaraborg Hospital, Skövde, Sweden; ^5^Department of Molecular and Clinical Medicine/Cardiology, Sahlgrenska Academy, University of Gothenburg, Gothenburg, Sweden

**Keywords:** Takotsubo syndrome, orexin, hypocretin, hypothalamus, COVID-19

## Abstract

Takotsubo syndrome (TTS) is an acute heart failure syndrome. Emotional or physical stressors are believed to precipitate TTS, while the pathophysiological mechanism is not yet completely understood. During the coronavirus disease (COVID-19) pandemic, an increased incidence of TTS has been reported in some countries; however, the precise pathophysiological mechanism for developing TTS with acute COVID-19 infection is unknown. Nevertheless, observing the symptoms of COVID-19 might lead to new perspectives in understanding TTS pathophysiology, as some of the symptoms of the COVID-19 infection could be assessed in the context of an orexin/hypocretin-system dysfunction. Orexin/hypocretin is a cardiorespiratory neuromodulator that acts on two orexin receptors widely distributed in the brain and peripheral tissues. In COVID-19 patients, autoantibodies against one of these orexin receptors have been reported. Orexin-system dysfunction affects a variety of systems in an organism. Here, we review the influence of orexin-system dysfunction on the cardiovascular system to propose its connection with TTS. We propose that orexin-system dysfunction is a potential novel explanation for the pathophysiology of TTS due to direct or indirect dynamics of orexin signaling, which could influence cardiac contractility. This is in line with the conceptualization of TTS as a cardiovascular syndrome rather than merely a cardiac abnormality or cardiomyopathy. To the best of our knowledge, this is the first publication to present a plausible connection between TTS and orexin-system dysfunction. We hope that this novel hypothesis will inspire comprehensive studies regarding orexin's role in TTS pathophysiology. Furthermore, confirmation of this plausible pathophysiological mechanism could contribute to the development of orexin-based therapeutics in the treatment and prevention of TTS.

## Introduction

Takotsubo syndrome (TTS) is an acute heart failure syndrome believed to be precipitated by emotional or physical stressors (1). However, the pathophysiological mechanisms underlying TTS are not yet completely understood (2). Among numerous possible explanations (1), “catecholamine-driven cardiac dysfunction remains the predominant hypothesis” (2). During the coronavirus disease (COVID-19) pandemic, an increased incidence of TTS has been reported in some countries (3). While the connection between TTS and COVID-19 remains unknown, this finding reveals a possible role of orexin/hypocretin in the development of TTS, as some of the symptoms of the COVID-19 infection could be assessed in the context of an orexin/hypocretin-system dysfunction (4).

A recent study demonstrated drastic increases in autoantibody reactivity in acute COVID-19 patients compared to uninfected controls. These findings included a high level of antibodies directed against the orexin receptor (5). Orexin/hypocretin “is a key component of the arousal system” (6), and the orexin/hypocretin system is associated with various neurocognitive, psychobiological, and physiological functions. The orexin-system plays an important role in central cardiovascular control and the regulation of cardiorespiratory function, and it seems to contribute to the response to stressors, especially psychogenic ones (7, 8). Additionally, orexin seems to ameliorate endothelial dysfunction (9). Therefore, we propose that orexin-system dysfunction may play a role in the development of TTS through several proposed pathophysiological mechanisms.

## Orexin/hypocretin system

Orexins are neuropeptides derived from hypothalamus (10). Orexin's circuits originate from the hypothalamus and project to various brain areas, including the locus coeruleus, thalamus, basal forebrain, tuberomammillary nucleus, cortex and others (6, 11). Orexins are cardiovascular neuropeptides (12), and lack of orexin may influence various pathophysiological mechanisms of cardiovascular diseases (13). Orexin seems to affect cardiovascular function due to “direct central sympathoexcitatory action” (7).

There are two isoforms of orexin: orexin-A and orexin-B (8). Orexin acts through its postsynaptic G-protein-coupled receptors (GPCRs): orexin type 1 (OX1R) and orexin type 2 (OX2R) receptors (6). Orexin-A acts on both receptors, while Orexin-B primarily on OX2R (7). Both OX1R and OX2R are “promiscuous in their signaling”, coupling three G-protein families: G_q_, G_s_, and G_i/o_ (14). Orexin receptors are expressed in a variety of organs in peripheral tissues, such as in the adrenal glands, gonads and the gut, indicating the existence of the peripheral orexin-system, as well as in the central nervous system (14–18). Although orexin is a cardiorespiratory neuromodulator (19), it belongs to a family of multifunctional neuropeptides and affects several functions of the nervous, cardiovascular, endocrine, respiratory, gastrointestinal, urinary, and reproductive systems (7, 19–21). For example, studies on orexin's endocrine function suggest it plays an important role in the regulation of hypothalamic-pituitary-adrenal (HPA), hypothalamic-pituitary-gonad (HPG), hypothalamic-pituitary-thyroid (HPT), growth hormone (GH), and prolactin axes (20).

## Dysfunction of the orexin-system

In narcolepsy with cataplexy, patients exhibit a profound loss of orexin neurons in the lateral hypothalamus (6). In addition, there are several other disorders and conditions that may be related to orexin-system dysfunction, including diabetes, obesity, cancer, neurogenic hypertension, obstructive sleep apnea, sudden infant death syndrome, neurodegenerative diseases and some neuropsychiatric disorders (4, 10, 15, 22).

The contribution of the two orexin receptors to orexin cardiovascular action is still unclear (7). In rats, the heart expresses both orexins and orexin receptors (23). Both OX1R and OX2R are likely involved in orexin's cardiovascular effects (7, 8). However, in rats OX1R was found significantly more in the hypothalamus than in the heart, while expression of OX2R was similar in both sites (23). Perez et al. found a greater expression of OX2R protein in the left ventricle than in the left atrium in diseased human myocardial tissue (24). OX2R is associated with left ventricular function and may have a role in the protective response to myocardial insults (24). Thus, OX2R could be a therapeutic target in cardiovascular diseases (25). OX2R agonism may improve myocardial function (i.e., better systolic function) and protects against chemical stress-induced ventricular dysfunction (24). Furthermore, orexin-A may be a biomarker that can predict left ventricular myocardial remodeling (26).

Little is known about orexin receptors' distribution in the heart; however, their presence in the left ventricle might represent a site of direct orexin action. Moreover, since the orexin-system “can act at all levels of the central autonomic network” (7), it may influence central cardiac function. Additionally, orexin neurons contain other modulators or neurotransmitters than orexin, including glutamate, dynorphin, galanin, and nitric oxide—substances whose exact roles remain only partially understood (27). The orexin-system might thus contribute to TTS without orexin playing a causative role.

## Possible role of orexin in pathophysiology of TTS

Recently, the working group of the European Society of Cardiology published a joint scientific statement regarding the pathophysiology of TTS (1, 28). Here, we reflect upon the proposed models and context that include a potential role for orexin-system dysfunction in developing TTS.

### A role for catecholamines and sympathetic activation

The role of catecholamines and the sympathetic nervous system (SNS) in TTS is well supported (1). Activation of the SNS by stressors may lead to an excess of catecholamines, which may, in turn, “have toxic effects on myocardial tissue” and manifest as transient left ventricle dysfunction (29). Orexin was shown to increase intracellular calcium (Ca), enhance epinephrine release from bovine adrenal medullary cells, and induce catecholamine synthesis (16). The central role in SNS regulation that the orexin-system seems to play, along with cardiac hypotrophy in orexin knockdown mice, suggests the orexin signal-pathway as a possible therapeutic target for cardiovascular diseases (30).

Findings from an orexin/ataxin-3 transgenic rat model (rats with a “minimal number of orexin neurons”), showed hypotrophic changes in the cardiac phenotype, suggesting the “involvement of the orexin-system in cardiac development” (31). Furthermore, those animals had “decreased responsiveness to the β-adrenergic blocker, propranolol”. Propranolol may reverse some actions following orexin-A administration (32) and is one of the drugs being considered for TTS management (33). The orexin-system contributes to cardiovascular and cardiorespiratory responses to certain stressors (7, 8). This response evoked by psychological stressors can be reduced by a blockade of orexin receptors (8). Thus, the β-adrenergic blockade may mediate orexin's influence on the cardiovascular response to stressors seen in TTS.

### G-protein coupled receptor kinase activity

The G-protein coupled receptor kinase (GRK) has a possible role in the physiopathology of TTS, with GRK2 and GRK5 isoforms being predominant in the heart (1). The regulatory phosphorylation site (Ser-262) “mutation of OX1R impairs its interaction with GRK2” (34), motivating further study in the TTS context. However, Ser-262 does not affect ORX-1R's “capability to interact with GRK5” (34).

### Ca^2+^ signaling and cell survival

A cyclic adenosine monophosphate (cAMP) formation and increased intracellular Ca concentration are common to cardiomyocyte responses to stress (35). Orexin receptors influence Ca^2+^ signaling (14). Depending on the milieu, “orexin receptor signals appear highly tunable”. For example, plasticity is observed in some cells upon longer-lasting stimulation, while others are stimulated to death (14). Orexin is necessary for the maintenance of fundamental membrane characteristics (36). As a result of orexin-A binding to OX1R, intracellular Ca increases, and the sodium/calcium exchange is activated (6). Increased activity of the orexin-system in the paraventricular nucleus activates calcium/calmodulin-dependent kinase II (CaMKII) expression, which may be important for the “downstream cardiovascular effects of CaMKII” (37). Independent of the diastolic or systolic levels of Ca, orexin-B causes an increase in contractile shortening in cardiomyocytes in rats (23). In addition, treatment with orexin-B results “in a dose-dependent increase in the myosin light chain and troponin-I (TnI) phosphorylation” (23).

Protein kinase B is important in the cell survival pathway, which is activated in the acute phase of TTS (38). Interestingly, orexin-A may affect cell proliferation in the insulin-secreting beta-cells via OX1R and the protein kinase B signaling pathway (39). Moreover, when human vascular endothelial cells are exposed to high glucose levels, orexin exerts potent protective effects (40). Similarly, orexin-B type 2 receptor agonist improved vascular function and myocardial injury induced by ischemia-reperfusion in diabetic rats (10). Taken together, orexin's anti-apoptotic and pro-survival actions (39) might be of interest to investigate in the context of TTS.

### Inflammatory response to injury

Injury that induces loss of neurons and cardiomyocytes results in the stimulation of inflammatory responses (35). Orexins have neuroprotective and immuno-regulatory properties and may have therapeutic potential for several inflammatory and neurodegenerative diseases, including Alzheimer's disease, cancers, inflammatory bowel diseases, multiple sclerosis, narcolepsy, obesity, and septic shock (41). A higher percentage of CD68^+^ macrophages and pro-inflammatory markers (HLA-DR and SOCS3) has been observed in TTS compared to control with no underlying heart conditions (42). These markers of inflammation might also be associated with the orexin-system (43–45). Influenza virus infection, as well as influenza vaccination, may be a trigger for TTS (38, 46). Influenza vaccine and infection with the H1N1 virus has been associated with narcolepsy with cataplexy—a condition characterized by profound loss of orexin neurons (6)—further supporting the possible role of the orexin-system in the pathophysiology of TTS.

### Central nervous system

TTS is a cardiovascular syndrome rather than solely a cardiomyopathy (28). The importance of the central nervous system in TTS is illustrated by a variety of psychiatric and somatic cerebral conditions that may trigger TTS (28). Based on previous research, Tsunematsu and Yamanaka suggest that “orexins stimulate sympathetic outflow under psychological conditions” (18). Functions of the cardiovascular system are modulated by orexins through “central autonomic control and peripheral actions” (35). In addition, orexin receptors' functional state may influence the responsiveness of central cardio-respiratory control mechanisms to adaptive stimuli (47). In the nucleus tractus solitarii, orexins elicit bi-directional dose-dependent cardiovascular (vasodepressor/vasopressor) effects (48). Furthermore, targeting OX2R in the brain may be a potential treatment of heart failure (49). Centrally administered orexin increases blood pressure, heart rate, respiratory amplitude and frequency, and sympathetic nerve activity; however, it is still unclear how the orexin receptors contribute to cardiovascular actions (7, 8).

### Biological characteristics

Nine of 10 patients with TTS are postmenopausal women (29); however, the reason for this phenomenon is not yet well-understood. During menopause, an exaggerated stress response can occur via an autonomic surge in the lack of estrogen (2). In female rats, orexin-A in the nucleus ambiguus elicited an “increase in vagal cardiomotor neuronal activity” in the heart, and estrogen may alter the sensitivity of neurons in this nucleus to orexin-A (50). Orexins could be the link between postmenopausal hypoestrogenism and increased cardiovascular risk (51). An increase in plasma orexin-A was found to parallel lower levels of estrogen in menopausal women (51). Additionally, higher levels of plasma orexin-A, associated with hypoestrogenism, may be partially reversed by estrogen-replacement therapy (52). Thus, El-Sedeek et al. suggested that estrogen's cardioprotective effect may be due to an inhibitory effect on orexin (52). In preclinical and clinical studies, sex differences in the orexin activities have also been reported (53). In addition, a study in women with gestational diabetes mellitus found that the level of orexin-A was lower compared to controls (54).

## Alternative explanatory framework for pathophysiology of TTS and orexin's role

**Figure 1:**
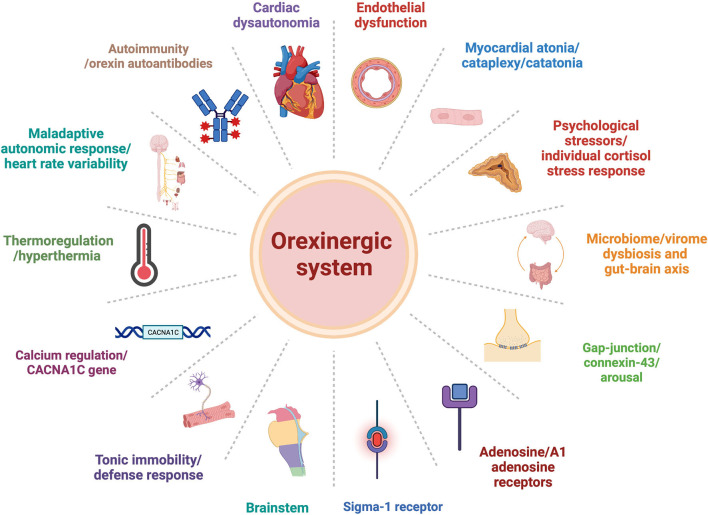
Orexinergic-system plausible central role within alternative explanation models of Takotsubo syndrome pathophysiology pathways.

Orexin may be involved in each of the proposed explanatory models presented above. Here, we would like to suggest some alternative mechanisms that may underlie TTS pathophysiology, and orexin's possible role in the process (Figure 1).

### Sigma-1 receptor and endothelial dysfunction

The activation of sigma-1 receptor (σ_1_R) may be clinically relevant to TTS (55). σ1R is widely expressed in a variety of tissues, including the brain and heart ventriculus (55). Inhibition of σ1R was shown to decrease severe acute respiratory syndrome coronavirus 2 (SARS-CoV-2) infection and viral replication in human iPSC-derived cardiomyocytes, while also compromising cytoskeleton integrity and leading to presumtive alteration in contractility (56). Coronary endothelial dysfunction has been recently proposed as the link between TTS and COVID-19 (57). In addition to the possible neuro- and cardio-protection provided by σ_1_R activation, different σ_1_R actions in the endothelium (58) may partly explain the proposed associations between TTS and COVID-19. In the context of proposed orexin-system dysfunction in TTS, σ_1_R plays a role in the response to activation of OX1R in the nucleus accumbens (59). More precisely, the antagonism of σ_1_R reduces the Ca^2+^ response elicited by selective OX1R agonist. It is possible that σ1R might play a significant role in TTS development, while the orexin-system might modulate this cascade mechanism.

### Brainstem

The sympathetic central nucleus and descending sympathetic nerves origins are both located in the brainstem (29). Neural changes in that area might be of importance in TTS. Furthermore, SARS-CoV-2 may invade the brainstem, disrupting how medullary centers function and leading to an increase in central sympathetic outflow (60). Due to orexin-system presence in the brainstem (6, 7, 61), and possibility that orexin potently influence cardiovascular function acting directly in that area (8), the orexin-system could play an important role in TTS pathophysiology through its influence on the brainstem.

### Adenosine/orexin interplay

To provide evidence for optimal pharmacological treatment for patients with TTS, a randomized registry clinical trial (BROKEN SWEDEHEART NCT04666454) is ongoing in Sweden, with adenosine as one of the interventions. Adenosine inhibits the activity of orexin neurons in the hypothalamus in a dose-dependent manner (62). Moreover, the A1 receptor exists on the cell bodies of orexin neurons, as well as on presynaptic terminals, thus innervating these neurons (62). Adenosine-mediated reduction in the excitatory afferents to the orexin-system considerably attenuates its excitability and limits the capability of the orexin-system to control its targets (62). A possible role of adenosine/orexin interplay in optimizing pharmacologic treatment for patients with TTS might be of interest to investigate.

### Psychological stressors and individual cortisol stress responses

Patients with TTS have high incidences of stressful life events (i.e., traumatic experiences) compared to healthy controls and patients with non-ST-segment elevation myocardial infarction (63). Thus, patients may potentially benefit from incorporating some recently proposed psychiatric interventions for stress-related disorders (64) into TTS treatment, in an attempt to prevent symptom progression. A recent meta-analysis revealed that hydrocortisone shows promising results in preventing post-traumatic stress disorder (PTSD) (65).

Given what we know about TTS and stress, it is pertinent to discuss the potential for nurturing the body's natural (HPA-axis) response immediately following the event that triggers TTS symptoms. Timing is critical in the treatment of stress-related disorders, as identical interventions may yield opposite results if given at different times (64). Furthermore, after a traumatic event, proper reactivity of the HPA-axis may be essential to spontaneous remission (64). It has been shown that a cortisol response in healthy men manifests with different response kinetics to repeated psychological stress, resulting in “low responders” and “high responders” (66). While some patients with TTS have shown a blunted cortisol stress response compared to healthy controls (63), findings regarding steroid replacement are conflicting (2). In some patients, cardiac dysfunction resolved with steroids, whereas in others, it worsened (2).

Ox2R mRNA in the adrenal cortex of male rats indicates orexin's involvement in the synthesis and/or release of adrenal steroids (17). Regarding the development of PTSD, low orexin tone during stressful events has been proposed to be associated with increased risk (67). Orexin's proposed role in TTS pathophysiology might be similarly understood. Therefore, orexinergic agents might be good candidates for treatment around the time of a triggering event.

### Microbiome/virome dysbiosis and gut-brain axis

SARS-CoV-2 driven immune dysregulation may provoke an imbalance in the microbiome/virome ecosystem (68). Developing a chronic disease may be due to microbiome/virome dysbiosis and dozens of chronic conditions are already connected with dysbiosis, including neuroinflammatory and metabolic disorders (68). Orexin has been suggested as one of the central mediators of “microbiota-gut-brain interactions during gastrointestinal inflammation” (69). TTS may be triggered by the gastrointestinal system, such as an exacerbation of inflammatory bowel diseases (38), and orexin may have therapeutic potential in these diseases (41).

### Gap-junction activation, connexin-43 expression, and arousal

Inflammatory activation may affect human cardiac fibroblasts, altering intracellular Ca^2+^ signaling and connexin 43 (Cx43) expression (70). Cx43 “is the most abundant gap-junctional protein” in the ventricular cardiomyocytes (71). Activation of sympatho-β-adrenergic receptors induces cardiomyocyte-derived interleukin (IL)-18 and stimulates Cx43 expression in fibroblasts (71). The orexin neurons in the lateral hypothalamic area may be silenced in the “knockout of the gap-junction subunit Cx43 in astrocytes” (72). Additionally, orexin-A may suppress the production of IL-18 (40). The interaction between orexin-system functioning, cardiomyocyte-derived IL-18, and Cx43 expression in cardiomyocytes/cardiac fibroblasts should be explored in future studies. Cx43 redistribution and gap-junction activation under restraint (immobilization) in rats, was shown to protect against lethal arrhythmias (73). Forced restraint induced catecholamine surge (73). As stated previously, an excess of catecholamines is a predominant theory of TTS etiology (2).

It has recently been proposed that TTS, excited delirium syndrome, and malignant catatonia can be categorized under the same framework of an extreme autonomic stress reaction (74). Under this framework, the authors suggest that these three conditions are all manifestations of a human form of capture myopathy. However, a clear explanation of the mutual pathophysiological mechanism has not yet been proposed. Because “orexin is a key component of the arousal system” (6), orexin-system dysfunction may be the common feature in all three conditions.

### Cardiac dysautonomia, autoimmunity, and norepinephrine reuptake

Dysautonomia is a result of a malfunction in the autonomic nervous system (75, 76). If cardiovascular homeostasis is influenced, then it is known as cardiac dysautonomia (75). It is still unclear if TTS belongs under the umbrella of cardiovascular autonomic dysfunctions or cardiac dysautonomia, or if orexin-system dysfunction may account for the development of the cardiac manifestation of dysautonomia. Orexin-A increases baroreflex sensitivity in rats (47), while orexin neurons in the perifornical area may shift baroreceptor reflex during defense response and mediate skeletal muscular vasodilation (77). Furthermore, OX1R antagonist may attenuate the redistribution of arterial blood flow during defense reactions in rabbits (78). Both TTS and postural orthostatic tachycardia syndrome (POTS) are associated with baroreflex dysfunction (79), and in some patients with TTS, “spontaneous baroreflex control of sympathetic tone” is decreased (38). One recent study aimed to measure G protein-coupled receptor autoantibodies in POTS. However, results from this study could not prove that patients with POTS and healthy controls had different autoantibody concentrations for several cardiovascular GPCRs (80). Autoimmunity has been suggested to play a potential role in the underlying pathophysiology of both POTS and TTS (80, 81). With that in mind, and in the context of orexin-system dysfunction, future studies should assess orexin autoantibodies in both TTS and POTS.

Presynaptic norepinephrine reuptake transporter (NET) deficiency leads to prolonged sympathetic activation, which might provide a genetic explanation for the pathophysiological mechanism in POTS (82). In contrast, administration of a norepinephrine reuptake inhibitor was shown to be more effective than a serotonin reuptake inhibitor in the treatment of cataplexy in mice (83). This finding suggests that NET could be a mediator through which the orexin-system might be responsible for the development of TTS and POTS.

### Maladaptive autonomic response and heart rate variability

Autonomic function might be observed by features of heart rate variability (HRV) (84). In the acute phase of TTS, HRV is reduced (79). A recent study by Evdokimov et al. showed that the majority of female inpatients with TTS had a vegetative imbalance (85). Results of HRV analysis in male rats suggested that sympatho-vagal imbalance might be related to orexin-A (86), thus contributing to the plausible role of orexin in developing TTS due to impaired sympatho-vagal interaction. In addition, a longitudinal population study found that in men, cardiovascular autonomic “differences in heart rate and blood pressure in late adolescence are associated” with subsequent major psychiatric disorders (87), a majority of which could be associated with orexin-system dysfunction. Furthermore, as HRV may give insight into the modulatory action of the cardiac autonomic nervous system (84), we suggest that depressed HRV may be a predictor of risk for TTS.

### Hyperthermia

Hyperthermia has been recently suggested as a possible trigger for TTS, via a rise in body temperature due to a catecholamine storm (88). We propose that orexin influences this cascade, as emerging evidence suggests it plays a role in thermoregulation (89) and in regulating the HPT-axis (20). Additionally, the “dramatic loss of estrogen tone during menopausal states” has been suggested to contribute to orexin-system hyperactivity, which may contribute to insomnia, anxiety, and more severe hot flashes (89). Patients with excited delirium syndrome, capture myopathy, and malignant catatonia—which, as noted earlier, may be viewed in the same framework as TTS—may suffer from hyperthermia (74). It has been suggested that orexin plays a role in thermoregulation during “stress related to exercise conditions” (90) and that orexin neurons orchestrate body temperature in a context-dependent manner (27). However, body temperature regulation via orexin neurons may be attributable to some of orexin's co-transmitters (27).

### Calcium regulation and the CACNA1C gene

Genetic variants of Ca-regulatory cardiac genes seem to predispose persons to TTS (91). *CACNA1C* might be one such gene, since its mutation may be implicated in the pathophysiology of early repolarization (92). *CACNA1C* mutation was also found in a patient with TTS and Timothy syndrome (93). Some sleep disorders, such as insomnia and narcolepsy, have also been associated with polymorphism of the *CACNA1C* gene (94). This gene encodes the alpha-1 subunit of L-type Ca^2+^ channels (Cav1.2), and Cav1.2 depletion seems to attenuate orexin's effects (94). *CACNA1C* gene mutation, the alteration of the Cav1.2 Ca-channels, and Ca-regulation might represent a pathophysiological pathway in the orexin-system's proposed influence on TTS development.

### Myocardial atonia, cataplexy, and catatonia

Muscle sympathetic nerve activity (MSNA) has been shown to vary with age and sex differences (95). In the context of TTS, findings related to MSNA are conflicting (96). During *cataplexy*, a significant increase in MSNA and blood pressure was observed along with a marked decrease in heart rate and irregular breathing pattern, mainly characterized by apnea (97). Furthermore, autonomic activation was observed after the cataplectic attacks, coinciding with muscle atonia. It is possible that the same mechanism, as in *cataplexy*, triggers the activation of cardiac sympathetic nerve activity and coincides with myocardial atonia. Further studies should explore whether the orexin-system may increase cardiac sympathetic nerve activity and whether simultaneous vasoconstrictions in microcirculation lead to cardiomyocyte damage.

*Cataplexy* may be elicited by vigorous exercise and strong emotions, both of which are associated with cardiovascular changes (98). These findings support the hypothesized role of *cataplexy* as homeostatic reflex (98), which might also be relevant for TTS. We could thus, consider TTS to be one form of a “prolonged *cataplexy* of the heart”, but also a “catatonic heart”. As previously mentioned, malignant catatonia and TTS might both be seen in the context of an extreme autonomic reaction (74). Interestingly, TTS and catatonia may simultaneously occur, such as in the setting of benzodiazepine withdrawal (99). Moreover, akinetic catatonia might be associated with glutamic acid decarboxylase 65 (GAD65) autoantibodies (100), and orexin neurons might activate the GAD65 network (101). Therefore, GAD65 might represent a point at which TTS, catatonia, and the orexin-system overlap.

### Tonic immobility and defense response

Tonic immobility (TI) represents “a response to fear or threat by remaining motionless”, coinciding with considerable changes in cardiac function (102). A recent study in rabbits demonstrated a significant decrease in heart rate, as well as changes in rhythm during TI. The same study demonstrated a significant increase in the diameter of the left ventricular chamber during systole, which consequently decreased fractional shortening and ejection fraction (102). Turner Giannico et al. note that while some researchers have suggested that TI has a biphasic response with the initial increase in sympathetic activity following an increase in parasympathetic activity, others have suggested the involvement of both the sympathetic and parasympathetic systems (i.e., the sympathovagal balance) (102).

Although the previously described changes of cardiac functions during TI have not been shown with the TTS phenotype in an animal model, the heart during death-feigning and TTS may share a common pathophysiology. Excitatory manipulation of the pathway (orexin neurons in the hypothalamus, noradrenergic neurons in the locus coeruleus, and the lateral amygdala) induces freezing (103). This finding might be important in the TTS context, as the role of orexin neurons in the *defense response* seems to be orchestration of the cardiovascular and respiratory systems, as well as thermoregulation depending on the context (27). Therefore, we suggest that TTS may be conceptualized as a *defense response* and a human cardiac expression of *tonic immobility* mediated by the orexin-system.

## Strength and limitations

The pathophysiological mechanism of TTS is not yet completely understood. In this paper, we propose orexin-system dysfunction as a new explanation model of TTS development. Yet, despite the appeal of this unique hypothesis, this article has several limitations. First, this was not a systematic literature review; therefore, subjectivity may have limited our selection of relevant evidence. Second, this paper was written from a clinical perspective, and while interdisciplinary, it did not include preclinical nor neuroscience experts, which would be necessary for a comprehensive analysis. Such collaboration is even more important given the complexity of this system and significant species differences in orexin physiology (104).

## Conclusions and implications

The current model explains TTS as a systemic rather than an exclusively cardiac reaction to stressors, and multitasking of the orexin-system might explain different TTS triggers. Furthermore, central and peripheral orexin-system networks, with involvement in a variety of pathophysiological mechanisms, could explain the complexity of TTS phenotype development as opposed to trying to identify anatomical structures that respond with TTS localization. Rather than having one potential pathophysiological mechanism, the orexin-system may be responsible for orchestrating different pathways that may, among others, evoke cardiotoxicity and manifest as TTS. We propose the orexin-system plays a role in the pathophysiology of TTS and hope to initiate studies aimed to test this hypothesis. If orexin-system dysfunction is associated with TTS development, then orexin-based therapeutics might be an option for prevention and treatment of TTS.

## Data availability statement

The original contributions presented in the study are included in the article, further inquiries can be directed to the corresponding author.

## Author contributions

RK conceived and explained the role of the orexin-system in the development of TTS and wrote this article's original manuscript after scientific discussion with MN and EO. MN and EO critically reviewed draft versions and continuously helped to theoretically shape the original hypothesis. Additionally, EO played a senior supervisory role. All authors accepted the final manuscript.

## Funding

This study was financed by grants from the Swedish state under the agreement between the Swedish government and the county councils, the ALF-agreement, the Swedish Heart-Lung Foundation and the Swedish Scientific Council.

## Conflict of interest

The authors declare that the research was conducted in the absence of any commercial or financial relationships that could be construed as a potential conflict of interest.

## Publisher's note

All claims expressed in this article are solely those of the authors and do not necessarily represent those of their affiliated organizations, or those of the publisher, the editors and the reviewers. Any product that may be evaluated in this article, or claim that may be made by its manufacturer, is not guaranteed or endorsed by the publisher.

## Abbreviations

OX1R: orexin type 1 receptor
OX2R: orexin type 2 receptor
TTS: Takotsubo syndrome.
